# An unusual groin mass. Seminal vesicle abscess: a case report

**DOI:** 10.1186/1757-1626-2-6531

**Published:** 2009-05-14

**Authors:** Sunita Saha, Georgina Wright, Tan Arulampalam, John Corr

**Affiliations:** 1 Department of General Surgery, Colchester General HospitalTurner Road, Colchester, Essex, CO4 5JUUK; 2 Department Laparoscopic and General Surgery, Colchester General HospitalTurner Road, Colchester, Essex, CO4 5JUUK; 3 Department of Urology, Colchester General HospitalTurner Road, Colchester, Essex, CO4 5JUUK

## Abstract

The rare pathology of seminal vesicle abscess is usually diagnosed with computerised tomography scan and confirmed with transrectal ultrasound. We report a recently encountered case where diagnosis proved difficult owing to the non-specific clinical presentation.

## Introduction

Seminal vesicle abscess is a rarely encountered pathology with only 28 reported cases in the English literature. Commonly reported presenting complaints include; fever, dysuria and other urogenital symptoms. Here we present a case of seminal vesicle abscess that we believe to be the first reported case presenting with a tender lump in the groin. We describe the diagnostic challenge of this unknown presentation and its subsequent management.

## Case presentation

We report a case of a 49 year old childless Caucasian male who presented with a one week history of a gradually enlarging, exquisitely tender groin mass. The patient denied systemic, bowel or genitourinary symptoms except on deeper questioning when a two week history of darkened urine was described. Past medical history included bilateral groin hernia repair as a child and an incision and drainage of perianal abscess one year before.

Clinical examination revealed a tender mass in the left groin extending into the left scrotum where a large cystic swelling was identified. A non-tender right testicle was palpated. The remainder of clinical examination including rectal examination was unremarkable.

Haematological indices demonstrated a mild neutrophilia and abnormal liver function tests. In view of these findings, a groin exploration was performed demonstrating a thick walled sac adherent to the vas and vessels. Surprisingly when incised, this drained >100ml of frank pus into which a corrugated drain was inserted. The pus later cultured coliform bacteria.

**Figure 1.**
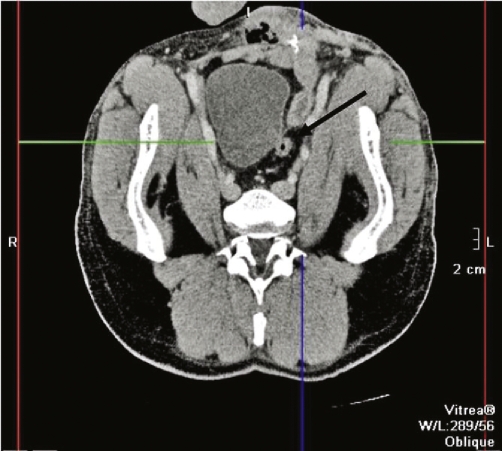
Transverse section magnetic resonance image demonstrating seminal vesicle abscess (arrowed).

Computerised tomography of the abdomen and pelvis revealed a thick-wall collection between the urinary bladder and rectosigmoid colon. In order to further delineate the extent of disease magnetic resonance imaging (Figure 1) was performed. This demonstrated a collection draining along the spermatic cord pointing to the left inguinal region.

In view of the likely urological source, a cystoscopy, rigid sigmoidoscopy to 15cm and laparoscopic drainage of the abscess were performed. Laparoscopic findings suggested the source to be the seminal vesicle; this was later confirmed by histological analysis.

A follow-up scan performed eleven days later confirmed successful drainage. Although there were no local complications, unfortunately the patient presented one month later with a pulmonary embolus and however made a good recovery.

## Discussion

Raijfer et al [1] first reported a case of seminal vesicle abscess in 1978 who presented with the classical features of fever and prostatic mass. Seminal vesicle abscesses are a rare entity with only a further 27 cases reported in the English literature [2-4]. A review by Pandey et al documented the clinical presentations of reported cases with 74% presenting with fever, 58% with dysuria and 32% with a tender prostate or ongoing epididymo-orchitis. The case described above exhibited none of the principal features described in this article [2]. A number of differential diagnoses exist for a tender groin mass including a groin abscess however to the authors knowledge this is the first documented case of the source being a seminal vesicle. Ryan and Harte [5] report a similar case of suppurative vastitis presenting as an inguinal mass with no involvement of the seminal vesicle.

Although the exact aetiology of seminal vesicle abscess is unclear, reported predisposing factors include urinary tract infection, diabetes, indwelling catheters, urological instrumentation/surgery, intraabdominal carcinomatosis and anatomical abnormalities [6-8]. Following eventual diagnosis, retrospective review of imaging suggested that the causative factor in this case was likely to have been a congenital anatomical anomaly of the ejaculatory duct. This presumption is supported by the fact that patient was subfertile.

In previous documented cases the most common causative organism is *Escherichia coli*. In fact, a study by Saglam et al. cultured *Escherichia coli* from the urine and abscess in 100% of cases [3]. This correlates with our findings.

Computerised tomography is the most frequently described diagnostic tool, although the most recent literature advocates the use of transrectal ultrasound [3]. The unusual nature of presentation in this case resulted in a delayed diagnosis which was made at the time of surgical exploration. Current recognised treatment modalities are; needle drainage via a transrectal or transperineal approach [6]. Laparoscopic drainage although not the recommended treatment, it proved to be effective in this instance.

Tender groin lumps are common in general surgical practice. We describe the first case of a seminal vesicle abscess being the underlying pathology.
